# The effect of Transcranial Direct Current Stimulation in addition to Tinnitus Retraining Therapy for treatment of chronic tinnitus patients: a study protocol for a double-blind controlled randomised trial

**DOI:** 10.1186/s13063-015-1041-2

**Published:** 2015-11-10

**Authors:** Sarah Rabau, Vincent Van Rompaey, Paul Van de Heyning

**Affiliations:** Faculty of Medicine and Health Sciences, Campus Drie Eiken, University of Antwerp, Antwerp, Belgium; University Department of Otorhinolaryngology and Head and Neck Surgery, Antwerp University Hospital, Wilrijkstraat 10, 2650 Edegem, Belgium

**Keywords:** Tinnitus retraining therapy, Transcranial direct current stimulation, Chronic tinnitus

## Abstract

**Background:**

Currently, there still is no treatment that eliminates tinnitus in all patients. Recent studies have shown that Tinnitus Retraining Therapy (TRT) significantly improves quality of life for tinnitus patients. Also, several studies have reported that transcranial Direct Current Stimulation (tDCS) has a positive effect on attention, working memory, long-term memory and other cognitive processes. The aim of this randomised placebo-controlled double-blind study is to evaluate the added effect of tDCS to TRT in chronic tinnitus patients. To our knowledge, this is the first study to combine both methods.

**Methods:**

Patients with chronic, non-pulsatile tinnitus will be randomised in two treatment groups: TRT and real tDCS versus TRT and sham tDCS. Evaluations will take place at baseline before therapy starts, at the end of the TRT and 3 months after therapy starts. The Tinnitus Functional Index will be used as the primary outcome measurement. Secondary outcome measurements will be the Visual Analogue Scale of Loudness, Hospital Anxiety and Depression Scale (HADS), Hyperacusis Questionnaire, psychoacoustic measurements and Event-related potential (ERP).

**Discussion:**

To our knowledge this is the first study to combine TRT and tDCS. The objective is to evaluate whether tDCS can provide faster and/or more relief from the annoyance experienced in chronic tinnitus patients’ daily lives. The advantage of the study is that it is double-blind and placebo-controlled.

**Trial registration:**

The present study protocol was registered on 31 October 2014 at Clinicaltrials.gov: NCT02285803.

## Background

Tinnitus is the perception of sound without the occurrence of a corresponding external sound source. Approximately 25.3 % of the US population experience some tinnitus and 7.9 % suffer from frequent tinnitus [1]. Currently, there is still no treatment that totally eliminates tinnitus in all patients. Therefore, the goal is to change the reaction to tinnitus and thus reduce the impact of tinnitus on the patient’s daily life. The type of counselling that tries to achieve this goal is called Tinnitus Retraining Therapy (TRT). Early studies have shown that 82 % respond positively to TRT [2]. TRT is comprised of two components: counselling and sound enrichment. Counselling is a one-on-one conversation between the patient and the audiologist or counsellor. A family member may be involved at the patient’s request. The counselling is based on the Jastreboff neurophysiological model [3]. Counselling will educate the patient on the working mechanism of the ear and tinnitus and promote adaptive coping behaviours. The second important aspect of TRT is sound enrichment. This can be achieved through a noise masker or even background noise in the environment. Sound enrichment results in partial or complete masking of the tinnitus, allowing the patient to ignore or transfer it to the subconscious. Philips et al. (2010) emphasised the importance of using low-level noise instead of completely masking the tinnitus to achieve down-regulation [4].

Transcranial Direct Current Stimulation (tDCS) is a form of neuromodulation in which a low current is applied to the brain by means of two electrodes: the anode placed on the dysfunctional site and the cathode placed on a ‘silent’ part of the body. Nowadays, mostly bifrontal tDCS is applied in tinnitus patients with the anode and cathode placed on the right and left dorsolateral prefrontal cortex (DLPFC) respectively. However, Martin et al. suggested placing the cathode on the right upper arm and the anode on the left DLPFC to prevent cathode-related inhibitory effects at the cortical site [5]. Research has shown that tDCS can provide temporary relief from tinnitus in 39.5 % of tinnitus patients [6]. In addition, previous studies have shown that tDCS has a beneficial effect on numerical competence [7] and working memory in depressed persons [8]. It also has also been reported beneficial to learning and memory in persons with a brain injury [9].

The aim of the present study is to evaluate the additive effect of tDCS to TRT. The idea is that tDCS would prime the central nervous system and thus create a stronger and faster learning effect for coping with tinnitus. This setup was based on the research of Martin et al. [10]. Their research showed that performance on a cognitive task could be improved by giving tDCS during the task [10]. The timing of tDCS was also found to be important. Furthermore, Martin et al. [11] found that tDCS simultaneously presented with a cognitive training task led to better within-session skill, with a significant difference found between conditions the following day when compared to presentation of tDCS prior to the cognitive training. Therefore, Martin et al. suggested the combination of tDCS with a rehabilitation protocol to improve the effect of the protocol [11].

## Methods

The present study is a randomised placebo-controlled double-blind trial in order to find out if tDCS adds value to TRT in chronic tinnitus patients. Patients will be referred by the ear, nose and throat (ENT) department of the Antwerp University Hospital and will be randomised in group A (real tDCS) or group B (sham tDCS).

### Ethics

Written consent will be obtained from every patient. The study protocol was approved by the ethical committee of the Antwerp University Hospital on 27 October 2014 with protocol number EC14/40/406 (Clinaltrials.gov: NCT02285803).

### Inclusion and exclusion criteria

The patient must meet specific criteria during consultation with the ENT before inclusion and randomisation. The appropriate examinations will be performed to make sure the patients meet the exclusion and inclusion criteria. During the study, the patient has the right to leave the study at any time. The occurrence of severe adverse events also can lead to discontinuation of the study.

The inclusion criteria are as follows:

- Duration of (worsening of) tinnitus between 3 months and 4 years
- Minimum age of the patient is 18 years old
- Maximum loudness of tinnitus on the Visual Analogue Scale (VAS) ≥ 4
- Tinnitus Functional Index (TFI) score ≥ 25

A patient is excluded from the study for the following reasons:

- Pulsatile tinnitus
- Auditory thresholds > 70 decibels hearing level (dBHL)
- Pregnancy
- Treatment of psychiatric disorder by a psychiatrist
- Pacemaker or defibrillator
- Acoustic neuroma
- History of cerebrovascular accident
- Ménière’s disease
- Whiplash
- Epilepsy
- Change of medication use

### Study protocol

#### Tinnitus Retraining Therapy

Every patient will receive approximately six sessions of TRT. The number of sessions will be adjusted to the needs of each patient. The TRT will consist of counselling and adjustment of a noise masker. The patient can try the noise masker at home for 3 months without purchase commitment. During counselling the patient will be educated on the working mechanism of the ear and tinnitus and also learn some coping strategies to deal better with sleeping and concentrations problems.

#### Transcranial Direct Current Stimulation (tDCS)

During every TRT session, the patient will receive tDCS during the counselling. There are two groups: one will receive ‘real’ tDCS and the other will receive ‘sham’ tDCS. The latter is the placebo group. Direct current is transferred by means of 2 saline-soaked pairs of surface sponge electrodes (35 cm^3^) and delivered by a specially developed, battery-driven, constant current neuroConn stimulator (neuroConn, Ilmenau, Germany) with a maximum output of 10 mA. The anode (positive electrode) will be placed on the right DLPFC (Brodmann area 9/46), and the cathode (negative electrode) will be placed on the left upper arm according to the 10–20 international system for electroencephalogram (EEG) electrode placement. The extracephalic placement of the cathode was chosen because of the reduced risk of current flowing from one electrode to the other, and as a consequence, the actual current could stimulate a deeper and wider brain area. Settings for both groups are described in Table 1. For the ‘real’ tDCS group, a constant current of 2 mA will be applied for 20 minutes with a fade-in and fade-out of 10 s. The ‘sham’ tDCS group will receive a tDCS session of only 10 s with a current of 1 mA. Patients in the sham group will experience the same tingling effect at the beginning of the tDCS as those in the real tDCS group, but no real stimulation will take place. Both the patient and investigator are blinded to the intervention type due to a five-digit code that can be entered in the study mode of the tDCS device that will be encoded sham or active tDCS stimulation. The five-digit codes were randomised and supervised by a third party.

**Table 1:** Settings for real Transcranial Direct Current Stimulation (tDCS) and sham tDCS

	Real tDCS	Sham tDCS
Current	2 mA	1 mA
Fade in/out	10 s	10 s
Duration	1200 s	10 s

The settings of tDCS are chosen so the patient would not notice a difference and they are blinded for the fact that they are randomised in the placebo or the real treatment group

#### Side effects

Possible side effects of tDCS are itching or tingling sensation, headache or fatigue and local burns at the position of the electrodes [12].

### Outcome measurements

There will be 3 test moments during the trial: before the start of therapy (T0), after the counselling sessions (T1) and after 84 days (T2). The primary outcome measurements will be assessed at every test moment. However, the secondary outcome measurements will only be assessed at T0 and T2 (Table 2). We are interested in the difference in the amount of change between both groups and the percentage of responders in each group.

**Table 2:** Timing of outcome measurements

		T_0_	T_1_	T_2_
Primary outcome measurements	TFI	x	x	x
Secondary outcome measurements	VAS	x	x	x
	HQ	x	x	x
	DS14	x		
	PGIC			x
	Tone audiometry	x		x
	Psychoacoustic measurements	x		x
	ERP	x		x

*DS14* 14-item questionnaire assessing for type D-personality, *HQ* Hyperacusis Questionnaire, *ERP* Event-related Potential, *PGIC* Patient Global Impression of Change, *TFI* Tinnitus Functional Index, *VAS* Visual Analogue Scale

In order to categorise our patient group, all subjects will undergo an audiometric hearing test according to current clinical standards (International Organisation for Standardisation (ISO) 8253–1:2010) with a 2-channel Interacoustics AC 40 (Interacoustics A/S, Middelfart, Denmark) in a soundproof audiometric booth. The TDH-39 headphone is used as transducer to measure air conduction thresholds of frequencies ranging from 125 Hz to 8 kHz. If thresholds exceeding 20 dBHL are found, bone conduction thresholds will be determined within a range of 250 Hz to 4 kHz.

### Primary outcome measurement

#### Tinnitus Functional Index (TFI)

The TFI is a self-reported questionnaire, consisting of 25 questions, which examines the impact of tinnitus on patients’ daily lives. The patient answers each question on a Likert scale ranging from 0 to 10. Questions 1 and 3 are expressed in percentages, and the Likert scale ranges from 0 % to 100 %. The total score is calculated with the mean of all questions. The answers are converted and the total score is expressed as a number between 0 and 100. In addition to the total score, the score of eight subscales can be determined. The subscales are the following: intrusiveness, reduced sense of control, cognitive interference, sleep disturbance, auditory difficulties attributed to tinnitus, interference with relaxation, reduced quality of life and emotional distress [13, 14].

### Secondary outcome measurements

#### Visual Analogue Scale (VAS) of loudness

The patient scores the mean and maximum loudness of their tinnitus on a scale of 0 (absence of tinnitus) to 10 (as loud as possible, cannot be any louder).

#### Hospital Anxiety and Depression Scale (HADS)

The HADS consists of 14 questions that assess anxiety and depression. The patient can choose between four answer options for each question. The score for both components is a summation of the scores of all the questions belonging to the subscale. A result greater than 8 suggests the presence of a depression and/or anxiety disorder [15, 16].

#### Hyperacusis Questionnaire (HQ)

The HQ is a 14-item questionnaire that surveys a patient’s hypersensitivity to sound. There are four answer options for every question: ‘no’, ‘yes a little’, ‘yes quite a lot’ and ‘yes a lot’. A score of 28 is the cut-off for auditory hypersensitivity [17, 18].

#### Patient Global Impression of Change (PGIC)

The PGIC assesses the patient’s perceived improvement in the severity of the tinnitus as a result of the therapy. The patient’s response options are the following: ‘very much improved’, ‘much improved’, ‘minimally improved’, ‘unchanged’, ‘minimally worse’, ‘much worse’ and ‘very much worse’.

#### DS14

The DS14 is a 14-item questionnaire that assesses the presence of a type-D personality. Half the items refer to negative affectivity and the other half refer to social inhibition. A score above or equal to 10 (range 0–28) on both scales classifies a person as a type-D personality [19]. The DS14 will be administered once at baseline before the start of therapy.

#### Psychoacoustic measurements

Psychoacoustic characteristics, such as frequency of tinnitus, loudness of tinnitus and minimal masking level (MML) will be determined. The frequency of tinnitus will be determined by means of frequency matching. The patient may choose between two presented tones or noises until a tone or noise is found that is similar to the patient’s tinnitus. In case of unilateral tinnitus, the contralateral ear is used as the test ear for frequency matching. In case of bilateral tinnitus, the tones will be presented in the ipsilateral ear. Furthermore, the loudness matching will be determined by comparison of the tinnitus to an external 1-kHz pure tone in the ipsilateral ear. Finally, the MML (i.e., the lowest level of broadband noise necessary to mask the tinnitus) will be determined in the ipsilateral ear.

#### ERP

The event-related potential (ERP) is used to assess the processing of sensory information. The ERP component P300 is elicited by the oddball paradigm whereby the patient has to count the number of rare stimuli (2 kHz) that are presented randomly between frequent stimuli (1 kHz) at an intensity of 70 decibels sound pressure level (dBSPL) with a ratio of respectively 20/80 %. The stimuli used is a tone-burst with rise/fall time of 10 ms and a plateau of 30 ms with a stimulus rate of 1.1/s. Silver/Silver Chloride electrodes are placed according to Standard International Electrode System rule 10–20 on the vertex (Cz), frontal (Fpz) and on the ipsilateral mastoid. The Bio-logic Navigator Pro (Natus, Pleasanton, USA) will be used to measure the ERPs.

### Statistical methods

Data will be analysed with SPSS statistical software version 20 (SPSS Inc., Chicago, IL, USA). Descriptive analyses as means and standard deviations will be used to describe the characteristics of the participants. To compare the results of both intervention arms, repeated measures will be computed if the data is normally distributed. If it is not, the data will be logarithmically transformed first. The outcome measurements will be set as dependent variables; the intervention group (sham versus real) and test moment (T0, T1, T2) are the independent variables. The interaction between test moment, intervention group and hearing thresholds on the outcome measurements will also be calculated. The significance level will be set at *p* < 0.05. Furthermore, the percentage of responders with a minimum reduction of 13 points on the TFI will be calculated by descriptive analyses and also the confidence interval of the observed difference.

### Sample size calculation

Sample size was calculated using Glimmpse 2.0.0, an open source online tool for calculating power and sample size. The selected statistical test was the univariate approach to repeated measures with Geisser-Greenhouse correction with interactions between test moment (T0, T1, T2) and group (real tDCS versus sham tDCS) and the TFI as outcome measurement. A reduction of 13 points is considered the minimum to obtain a meaningful reduction in TFI outcome scores. To measure improvement, the effect size of the TFI is moderate to high [13]. Based on this information, an overall sample size of 24 was needed to reach a desired power of 0.8, assuming a type-I error rate of 0.05 and a standard deviation of 20.

### Dissemination protocol

According to the Standard Protocol Items: Recommendations for Interventional Trials (SPIRIT) guidelines, the authors declare that data that break the blind will not be presented prior to release of mainline results. Breaking of the blind will occur at the end of the study. A clinical article will be written on the primary and secondary outcomes of the study and will be disseminated regardless of the magnitude or direction of effect. The present trial is not industry initiated; therefore, there are no publication restrictions imposed by sponsors. In addition, a full study report, anonymised participant-level dataset and statistical code for generating the results will be made publicly available no later than 3 years after termination of the study for sharing purposes.

## Discussion

Currently, there is no specific treatment to cure tinnitus in all patients. Although tDCS is a known treatment for tinnitus, it has shown only limited relief in 39.5 % of patients [6]. However, previous studies have shown that TRT results in tinnitus relief for 82 % of the study population [2]. This is a long-term process, and our goal of this study is to evaluate if tDCS can provide faster and/or more relief of the annoyance experienced in patients’ daily lives.

The advantage of the study is that it is double-blind and placebo-controlled. The fact that we compare sham and real tDCS will rule out the placebo effect of tDCS. The study is also based on the literature: a recent study showed that tDCS presented simultaneously with a cognitive training task led to better within-session skill, with a significant difference found between conditions the following day when compared to presentation of tDCS prior to the cognitive training. The authors suggested combining tDCS with rehabilitation protocols [11]. To our knowledge, this is the first study to combine both study methods.

### Trial status

To date, patient recruiting has started and the first patients will be randomised in the near future. The end date of study will be December 2015.

## Abbreviations

dBHL: decibels hearing level
dBSPL: decibels sound pressure level
DLPFC: dorsolateral prefrontal cortex
EEG: electroencephalogram
ENT: ear, nose and throat
ERP: Event-related potential
HADS: Hospital Anxiety and Depression Scale
HQ: Hyperacusis Questionnaire
ISO: International Organisation for Standardisation
MML: minimal masking level
PGIC: Patient Global Impression of Change
SPIRIT: Standard Protocol Items: Recommendations for Interventional Trials
tDCS: Transcranial Direct Current Stimulation
TFI: Tinnitus Functional Index
TRT: Tinnitus Retraining Therapy
VAS: Visual Analogue Scale

## References

[1] Shargorodsky J, Curhan GC, Farwell WR (2010). Prevalence and characteristics of tinnitus among US adults. Am J Med.

[2] Herraiz C, Hernandez FJ, Plaza G (2005). Long-term clinical trial of tinnitus retraining therapy. Otolaryngol Head Neck Surg.

[3] Jastreboff PJ, Hazell JW (1993). A neurophysiological approach to tinnitus: clinical implications. Br J Audiol.

[4] Phillips JS, McFerran D. Neurophysiological model-based treatments for tinnitus (Protocol). Cochrane Database Syst Rev. 2010;(1). doi:10.1002/14651858.CD008248.

[5] Martin DM, Alonzo A, Mitchell PB, Sachdev P, Galvez V, Loo CK (2011). Fronto-extracephalic transcranial direct current stimulation as a treatment for major depression: an open-label pilot study. J Affect Disord.

[6] Song JJ, Vanneste S, Van de Heyning P, De Ridder D (2012). Transcranial direct current stimulation in tinnitus patients: a systemic review and meta-analysis. ScientificWorldJournal.

[7] Cohen Kadosh R, Soskic S, Iuculano T, Kanai R, Walsh V (2010). Modulating neuronal activity produces specific and long-lasting changes in numerical competence. Curr Biol.

[8] Oliveira JF, Zanao TA, Valiengo L, Lotufo PA, Bensenor IM, Fregni F (2013). Acute working memory improvement after tDCS in antidepressant-free patients with major depressive disorder. Neurosci Lett.

[9] Polanowska K, Seniow J (2010). Influence of transcranial direct current stimulation on cognitive functioning of patients with brain injury. Neurol Neurochir Pol.

[10] Martin DM, Liu R, Alonzo A, Green M, Player MJ, Sachdev P (2013). Can transcranial direct current stimulation enhance outcomes from cognitive training? A randomized controlled trial in healthy participants. Int J Neuropsychopharmacol.

[11] Martin DM, Liu R, Alonzo A, Green M, Loo CK (2014). Use of transcranial direct current stimulation (tDCS) to enhance cognitive training: effect of timing of stimulation. Exp Brain Res.

[12] Poreisz C, Boros K, Antal A, Paulus W (2007). Safety aspects of transcranial direct current stimulation concerning healthy subjects and patients. Brain Res Bull.

[13] Meikle MB, Henry JA, Griest SE, Stewart BJ, Abrams HB, McArdle R (2012). The tinnitus functional index: development of a new clinical measure for chronic, intrusive tinnitus. Ear Hear.

[14] Rabau S, Wouters K, Van de Heyning P. Validation and translation of the Dutch version of the Tinnitus Functional Index. B-Ent. 2014;10:251–58. 25654947

[15] Zigmond AS, Snaith RP (1983). The hospital anxiety and depression scale. Acta Psychiatr Scand.

[16] Spinhoven P, Ormel J, Sloekers PP, Kempen GI, Speckens AE, Van Hemert AM (1997). A validation study of the Hospital Anxiety and Depression Scale (HADS) in different groups of Dutch subjects. Psychol Med.

[17] Khalfa S, Dubal S, Veuillet E, Perez-Diaz F, Jouvent R, Collet L (2002). Psychometric normalization of a hyperacusis questionnaire. ORL J Otorhinolaryngol Relat Spec.

[18] Meeus OM, Spaepen M, Ridder DD, Heyning PH (2010). Correlation between hyperacusis measurements in daily ENT practice. Int J Audiol.

[19] Denollet J (2005). DS14: standard assessment of negative affectivity, social inhibition, and Type D personality. Psychosom Med.

